# Clinical and immunological spectra of human cutaneous leishmaniasis in North Africa and French Guiana

**DOI:** 10.3389/fimmu.2023.1134020

**Published:** 2023-07-27

**Authors:** Nasreddine Saidi, Romain Blaizot, Ghislaine Prévot, Karim Aoun, Magalie Demar, Pierre André Cazenave, Aida Bouratbine, Sylviane Pied

**Affiliations:** ^1^ Univ. Lille, Univ. French Guiana, CNRS UMR 9017-INSERM U1019, Center for Infection and Immunity of Lille-CIIL, Institut Pasteur de Lille, Lille, France; ^2^ Laboratoire de Recherche, LR 16-IPT-06, Parasitoses Médicales, Biotechnologies et Biomolécules, Institut Pasteur de Tunis, Université Tunis El-Manar, Tunis, Tunisia; ^3^ Centre National de Référence des Leishmanioses, Laboratoire Associé, Hôpital Andrée Rosemon, Cayenne, French Guiana, France; ^4^ Service de Dermatologie, Hôpital de Cayenne, Cayenne, French Guiana, France; ^5^ Service de Parasitologie-Mycologie, Institut Pasteur de Tunis, Tunis, Tunisia

**Keywords:** *L. major*, *L. infantum*, *L. tropica*, *L. guyanensis*, *L. braziliensis*, cutaneous leishmaniasis (CL), clinical manifestation, local immune response

## Abstract

Cutaneous leishmaniasis (CL) caused by infection with the parasite *Leishmania* exhibits a large spectrum of clinical manifestations ranging from single healing to severe chronic lesions with the manifestation of resistance or not to treatment. Depending on the specie and multiple environmental parameters, the evolution of lesions is determined by a complex interaction between parasite factors and the early immune responses triggered, including innate and adaptive mechanisms. Moreover, lesion resolution requires parasite control as well as modulation of the pathologic local inflammation responses and the initiation of wound healing responses. Here, we have summarized recent advances in understanding the *in situ* immune response to cutaneous leishmaniasis: *i*) in North Africa caused by *Leishmania (L.) major*, *L. tropica*, and *L. infantum*, which caused in most cases localized autoresolutives forms, and *ii*) in French Guiana resulting from *L. guyanensis* and *L. braziliensis*, two of the most prevalent strains that may induce potentially mucosal forms of the disease. This review will allow a better understanding of local immune parameters, including cellular and cytokines release in the lesion, that controls infection and/or protect against the pathogenesis in new world compared to old world CL.

## Introduction

Leishmaniasis is a parasitic disease caused by a vector-borne protozoan parasite belonging to the *Leishmania* genus. It is transmitted as a flagellated promastigote *via* the bite of an infected sandfly (1, 2). Following its inoculation, promastigotes are ingested by innate cells mainly macrophages, neutrophils, and dendritic cells, where they evolve into amastigotes. *Leishmania* parasite are distributed in more than 98 countries, with 350 million people exposed and 12 million infected (3, 4). The cutaneous form is the most frequent, with an estimation of 600 000 to 1 million new cutaneous leishmaniasis (CL) cases per year. This disease is also responsible for significant psychosocial impacts due to the scars that persist after recovery and the associated social stigma (5). In the Old World, specifically in North Africa (NA), CL is caused by three species: *L. major*, *L. tropica*, and *L. infantum*. Cutaneous leishmaniasis is highly prevalent in Morocco, Algeria, and Tunisia. In the New World (NW), CL is widely distributed in South and Central America. It is mainly caused by *L. amazonensis*, *L. guyanensis*, *L. panamensis*, *L. peruviana*, *L. mexicana*, and *L. braziliensis* (6). These species can cause cutaneous and sometimes mucosal leishmaniasis (ML), with only a small potential for self-healing. French Guiana (FG) is a good example of an endemic South American country, where numerous works have been published on CL. The disease is basically divided into four clinical phenotypes (1): Localized CL (LCL) (2), mucocutaneous leishmaniasis, and (3) diffuse and (4) disseminated CL (7–9). Skin lesions result from a deregulated immune response which is unable to eliminate the intracellular parasites. This could explain the high number of parasites in the inflammatory infected zone for some forms of CL (10). Unbalanced T helper (Th)1/Th2 responses has been associated with an increased tissue destruction and a worsening of skin lesions. Moreover, in a susceptible murine model of *L. major* infection, improved resistance to infection was linked to the production of cytokines in lymph nodes. In most mouse strains, Th1 cells bring resistance through the secretion of IFN-γ. In contrast, susceptible mice generate a Th2 cell response characterized by the production of IL-4 and IL-13, which hampers the ability of IFN-γ to trigger toxic metabolites (11). The evolution of lesions is determined by a complex interaction between many factors triggered by the early immune responses, including innate and acquired immune mechanisms (10). However, there is a significant lack of information on the cutaneous immune response within CL lesions determining the evolution of the disease both in NA and FG. A better knowledge of the pattern of immune mechanisms and related factors involved in lesion progression or healing might provide helpful information for identifying new immunotherapeutic targets and new drugs.

In this review, we will sum up the current knowledge on clinical manifestations, lesion evolution and local immune response associated with *L. infantum*, *L. major* and *L. tropica* in NA and *L. guyanensis* and *L. braziliensis* CL in FG. We will also discuss similarities and divergences in immune mechanisms induced in NA and FG.

## Comparison of clinical manifestations and disease outcome between North Africa and French Guiana

**Figure 1:**
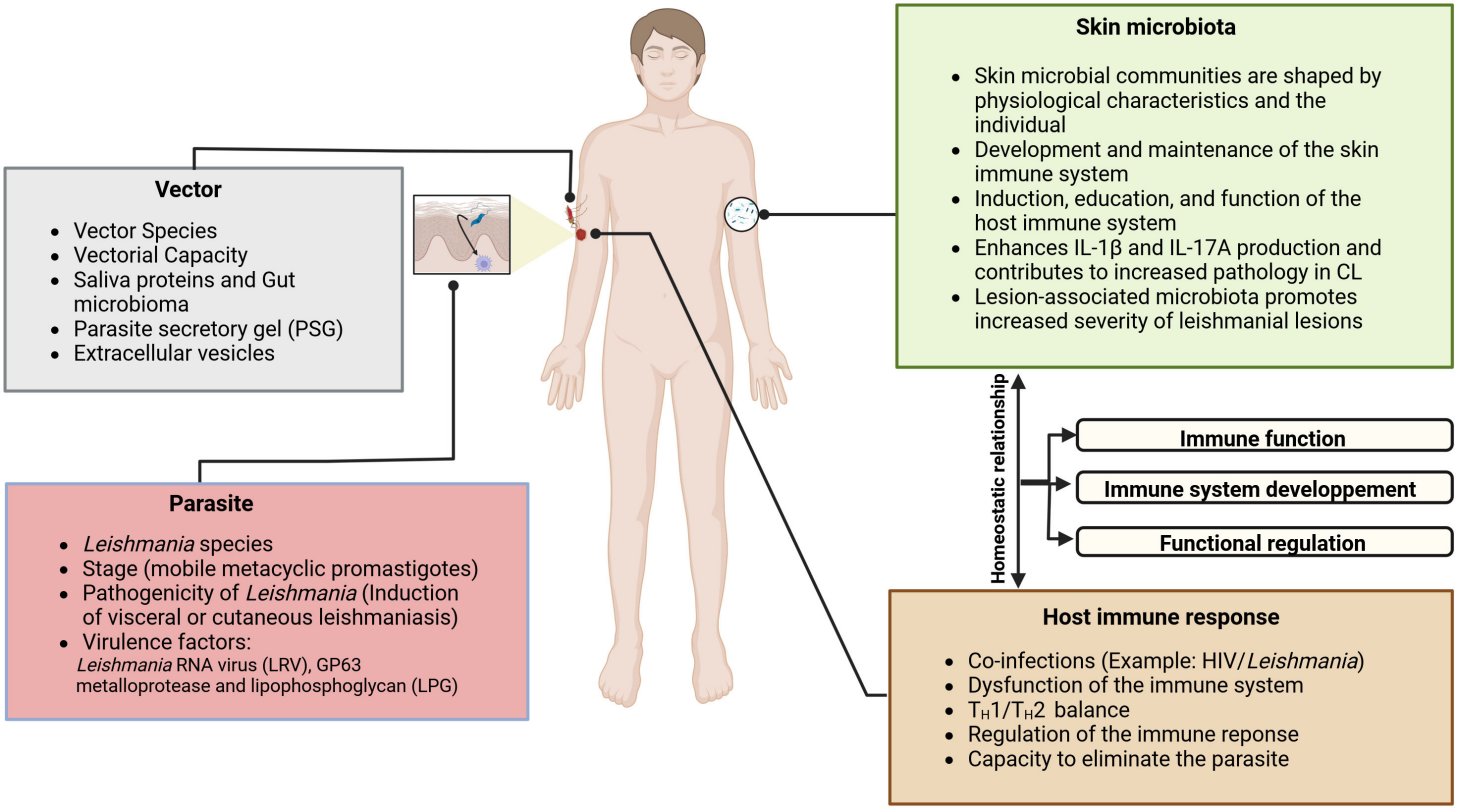
The pathogenesis of CL and the evolution of lesions are multifactorial; it depends on the complex interactions between the *Leishmania* parasite, the immune system, and the skin environment, including the vector-injected particles and the microbial skin communities. Sandfly saliva contains potent vasodilators, maxadilan, and adenosine, described respectively in *Lutzomyia longipalpis* and *P. papatasi*, that prevent clotting at the biting site (12), in addition of proteins that trigger a host immune response (13–15). As clearly demonstrated by several investigators, sandfly saliva contains immunomodulatory molecules that have been shown to enhance disease progression (16–18). Some of them, such as the parasite secretory gel (PSG), afford *Leishmania* protection from a hostile pro-inflammatory environment. They may directly regulate macrophages activation in dampening the early pro-inflammatory response and orchestrating wound repair and re-epithelialization (19). In fresh wounds, a robust pro-inflammatory response is required to sterilize the damaged tissue of potentially pathogenic bacteria. The skin microbiota plays a fundamental role in the host immune system’s induction, education, and function. In turn, the host immune system has evolved multiple means to maintain its homeostatic relationship with the microbiota. It has been shown that *Staphylococcus* spp., *Streptococcus* spp., *Enterococcus spp*, *Pseudomonas spp*, and other opportunistic bacteria are present in CL lesions (20–22). It was proven using a *Leishmania*-infected mouse with dysbiotic skin microbiota that naturally acquired dysbiosis can cause a change in inflammatory responses and disease progression. It has been demonstrated that the skin microbiome could modulate the skin’s immune response by enhancing IL17 production, which is essential in mediating inflammation in CL. While Th17 cells are a source of IL-17, it is possible that IL-17 produced by innate lymphoid cells present in the skin could contribute to disease progression. In effect, Scoot’s team has suggested that following infection by *L. major*, RORγt+ILCs produced IL-17 in the skin may contribute to disease progression.

The clinical presentation and the outcome of CL depend on multiple factors, including species involved, lesion location, sandfly infectivity, comorbidities, treatment modalities, skin microbiota and host immune responses against *Leishmania* (**Figure 1**) (23–25). The clinical appearance of CL is initially characterized by an erythematous, non-specific papule or patch following an incubation period after the bite of the *Leishmania* infected sandfly. This sub-clinical or early CL has the potential for self-healing; it then evolves into ulcerated (85%) or non-ulcerated (nodules, papules, plaques 15%) specific lesions. Some lesions have a very small potential for self-healing, especially those in the New World, and almost always require a treatment; the median incubation of CL in French Guiana is 25 days. Hypopigmented, hyperpigmented or atrophic scars can persist after treatment, depending on the skin phototype (26–31). In some cases, the lesions can persist for more than one year and become a chronic non-healing form (32, 33). Therefore, some infected patients leading to disfigured (34–36). However, the disease outcome also depends on the parasite species (**Table 1**).

**Table 1:** Summary of the different *Leishmania* species, clinical presentations, vector and transmission cycles in North African countries (Old World) and French Guiana (New World).

*Leishmania* species	Clinical presentation	Vectors	Transmission cycle	References
***L. major***	Localized	*P. papatasi*	Zoonotic	(9, 37–39)
***L. infantum***	Localized Visceral Leishmaniasis	*P. perfiliewi* *P. longicuspis* *P. ariasi*	Zoonotic	(9, 37, 38)
***L. tropica***	Localized	*P. sergenti*	Zoonotic	(9, 40, 41)
***L. guyanensis***	Single and multiple skin lesions Rare proportion of mucocutaneous cases Disseminated	*Lutzomyia (Ny.) umbratilis*	Zoonotic	(42–46)
***L. braziliensis***	Disseminated Important proportion of mucocutaneous cases	*Lutzomyia wellcomei* *Lu. (Ny.) neivai* *Lu. (Ny.) whitmani* *Lutzomyia ovallesi*	Zoonotic	(45, 47–50)

**Figure 2:**
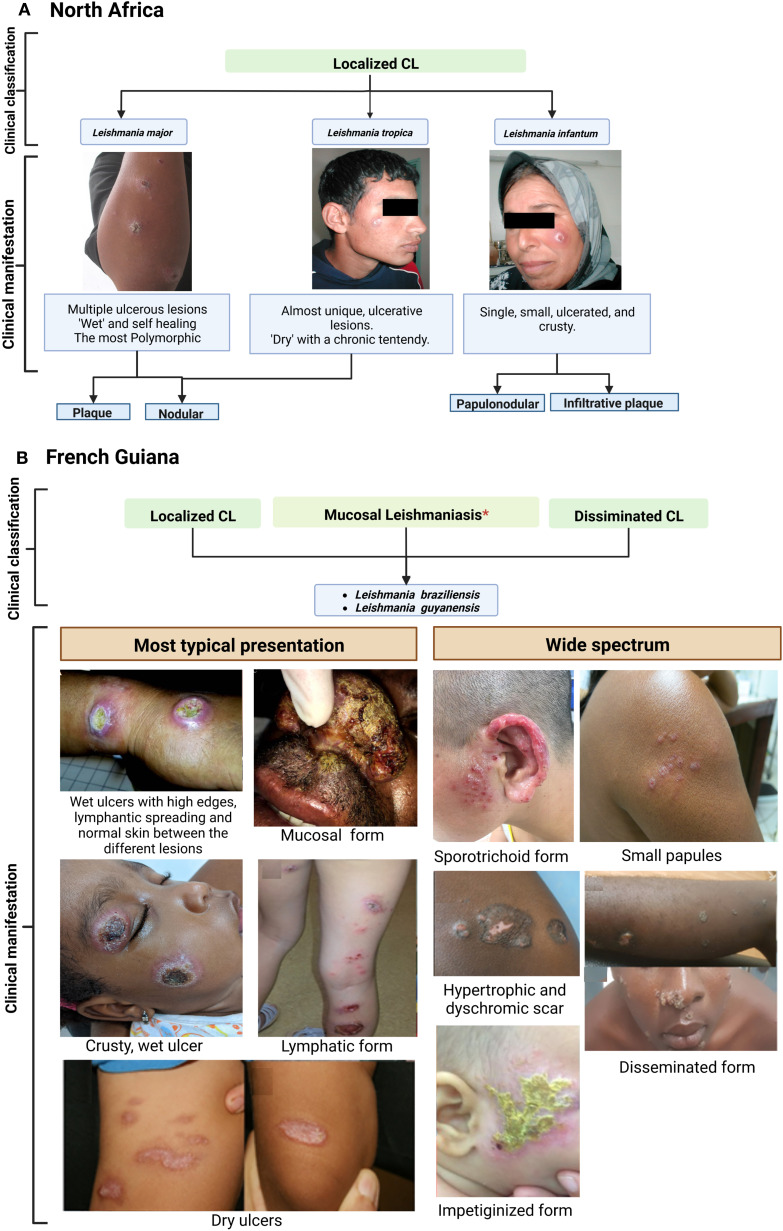
Clinical features of cutaneous leishmaniasis caused by *L. major*, *L. tropica* and *L. infantum* (Old world) **(A)** compared to *L. braziliensis* and *L. guyanensis* (New World) **(B)**. Case photos were provided by the laboratory of Medical Parasitology and Mycology. Institute Pasteur of Tunis. and Dermatology Department, Centre Hospitalier de Cayenne, French Guiana. ^*^Risk for mucosal involvement is about 6% for *L. braziliensis* and 1% for *L. guyanensis*. Reference: North Africa; French Guiana (9, 53–57).

Concerning *L. major*, this species is the most frequent in NA countries, where it remains a major public health problem, with more than 1000 to 8 000 cases yearly in Tunisia and 10,000 to 25,000 in Algeria (9, 40, 51, 52). Lesion can be single or multiple, usually with ulcerative nodules. These primary lesions can give birth to secondary peripheral lesions “satellite” (**Figure 2**) (9, 58). The most common type of ulcer is the ulcero-crusted form (59), also known as the “wet” or “rural” type. It is characterized by a painless ulcer with a clear raised border and a brownish scab covering it, commonly affecting the upper and lower limbs. CL caused by *L. major* has a wide range of clinical presentations, so it should be considered as a possibility in many skin diseases, including actinomycetoma, pyoderma gangrenosum, and various types of skin cancer. In Tunisia, about 11 uncommon clinical forms of CL due to *L. major* have been reported in zoonotic foci in Central and South regions of the country (60–62). This diversity may be due to the combination of the parasite’s genetics and the host’s immune response.

*L. tropica*, is responsible for chronic CL (CCL). Among the different countries of NA, Morocco presents the widest endemic areas and the highest incidence for this species (9). Compared with *L. major*, cutaneous lesions are distinguished by their chronicity; their appearance is dry and slightly inflamed. Therefore, this makes *L. tropica* CL more difficult to diagnose, which causes a delay in patient care (9, 63–66). Lesions in CCL are drier than in *L. major*, with papules, nodules, or crusty ulcers. Also, they are different in location and size compared to other species, as it mainly appears on the face and are typically smaller, with a diameter of less than 2 cm (41, 51, 67, 68).

The third CL causative species in NA is *L. infantum*, the primary responsible agent of visceral leishmaniasis (VL) (69). Between 50 and 150 cases are reported annually in Tunisia; few data are available for other countries (9). Compared to the previous two, the clinical appearance of wounds is more significant (58, 70). The duration between the bite of the infective sandfly and the onset of symptoms may vary between several weeks to 1 year (average 3-6 months) (51, 71). Another characteristic is that lesions may last longer than those with *L. major*, persisting for several months or years; such lesions are often slow to heal and may leave large, disfiguring, or disabling scars. Clinically, *L. infantum* lesions may present as papules or nodules, typically on the extremities and bordered by a large zone of infiltration (9, 37). Plaque is the most frequent clinical manifestation of cutaneous *L. infantum* involvement, followed by ulcers, nodules, and papules (72). The clinical presentation is a single small plaque on the face (9). Cases of mucosal involvement have recently been reported in travelers returning to Europe but are seldom described by North African teams (73).

In French Guiana, CL is caused by certain species belonging to the *Leishmania Viannia* subgenus, which is endemic to Central and South America (74). *L. guyanensis* represents more than 80% of cases in FG, while *L. braziliensis* has emerged in the 2000s and represents about 10-15% of cases (75). Other species, such as *L. lainsoni*, *L. naiffi*, and *L. amazonensis* can also be found but remain rare and are not part of this review (76). Though a complex clinical classification has not been proposed in FG, a very wide spectrum of clinical lesions can be observed, including impetigo-like, lupoid-like, sporotrichoid, crusted ulcers, patches, plaques, nodules, papules, or even extensive sores (**Figure 2**). These atypical forms have also been described in other countries of South America, notably Brazil (77, 78). However, the typical form of CL in French Guiana and the rest of the Amazon basin consists of a wet, acute ulcer which is slightly painful and usually without secondary bacterial infection (44). Several lesions can be found, particularly when *L. guyanensis* is involved. The skin between the lesions is normal, without erythema. A lymphangitis with or without parasitic nodules can be observed. Compared to Old World (OW) leishmaniasis, New World (NW) forms are known for a greater tendency to disseminate systemically in the skin (6, 79).

In addition, a great number of lesions with lymphatic involvement are observed for *L. guyanensis* CL in FG (42, 43) (**Figure 2**). This species is known to occasionally cause mucosal issues, but it is less common than mucosal disease caused by *L. braziliensis* (44, 80–82). Indeed, less than 1% of *L. guyanensis* cases in FG display a mucosal involvement (81, 83). *L. braziliensis* CL can manifest in different ways, from a single ulcer on the skin, which is often associated with satellite adenomegaly, to the less frequently disseminated form (82, 84). Potentially severe mucosal involvement of the upper airways are typically described with this species (47, 82, 85–87). Single lesions are more frequent than with *L. guyanensis* (44, 83).

## Cell-mediated immune responses associated with protection in the skin of CL patients

**Figure 3:**
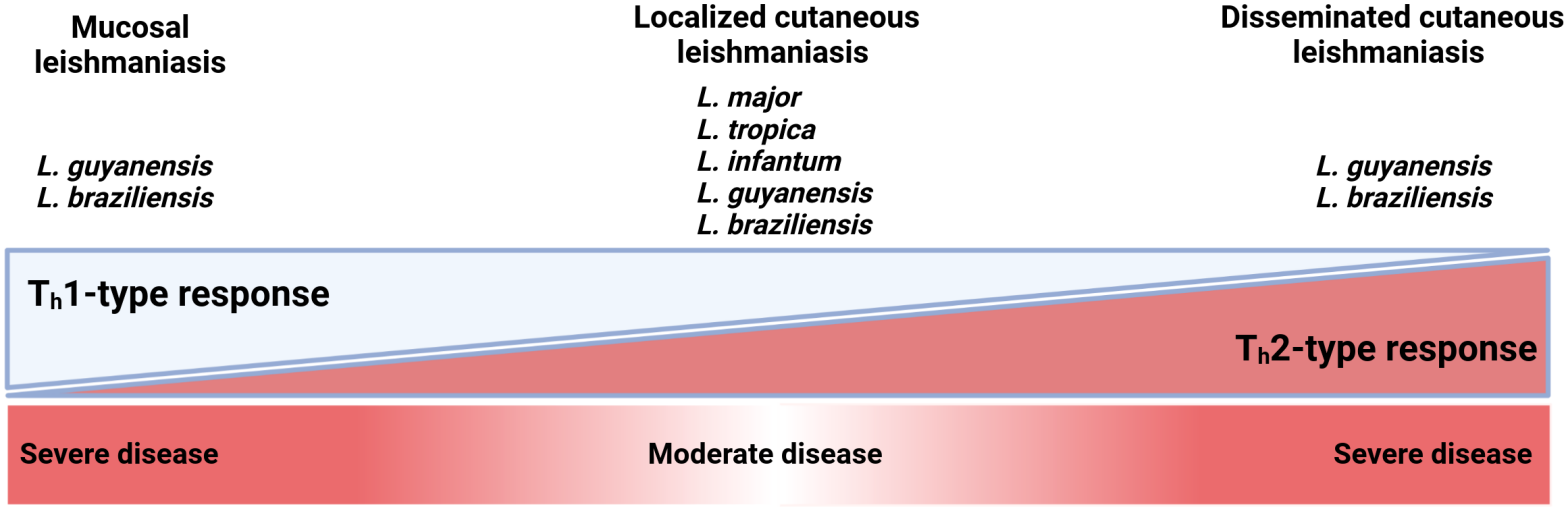
The T helper 1 (Th1)-type T helper 2 (Th2)-type balance across the cutaneous leishmaniasis severity. Mucosal and disseminated CL caused by *L. guyanensis* and *L. braziliensis* are the most severe form of the disease. They are on opposite sides of the Th1 and Th2 response, which enhances disease severity in an exaggerated response. An uncontrolled Th1 response in mucocutaneous leishmaniasis can cause an exaggerated cellular response, in which *Leishmania* amastigotes spread to the nasopharyngeal mucosa and cause tissue damage resulting in disfiguring lesions. For localized cutaneous leishmaniasis, mixed T_H_1 and T_H_2 responses have been observed during the active stage of infection; the Th1 profile is mainly associated with the healing of lesions.

**Figure 4:**
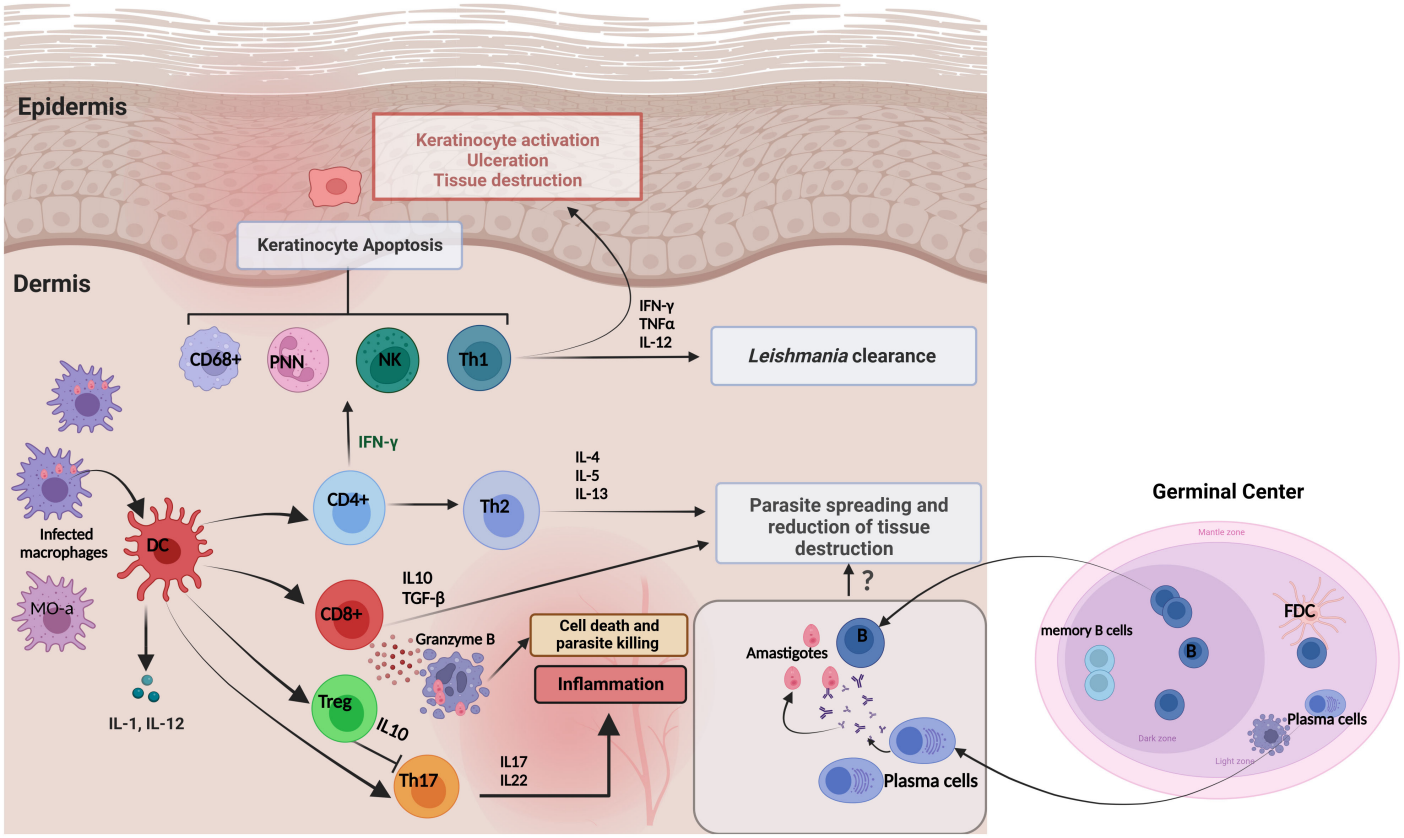
Immune responses and skin cytokine profile during cutaneous leishmaniasis infection. Pro-inflammatory cytokines are produced primarily to amplify the immune response to *Leishmania* infection. The major proinflammatory cytokines include TNF-α, IFN-γ, IL-1, IL-8, IL-12, IL-17 and Granzyme β. In contrast, anti-inflammatory cytokines are immunoregulatory molecules that counteract the effects of pro-inflammatory cytokines to limit the inflammation. These major anti-inflammatory cytokines include IL-5, IL-6, IL-4, IL-10, IL-13, and TGF-β. It has been proposed that the existence of Tregs in infected tissues could be an immune response from the host to maintain the balance to control Leishmania infection and reduce excessive inflammation that supports parasite survival. This is achieved by Tregs inhibiting Th17 cells through the production of IL-10. The role of the local humoral response to amastigote spreading and tissue destruction is still uncompletely understood.

CL in NA and FG is characterized by various immunological features (**Figure 3**) (44, 47, 58, 74, 88–90). Once the *Leishmania* infected sandfly bites, the infection starts with an asymptomatic “silent phase” of variable duration, characterized by an inflammatory wave of poly morpho nuclear (PMN), dendritic cells (DC), and monocyte-derived macrophages which harbour a proliferation of amastigotes intracellular parasites (10, 91, 92). Then, a massive recruitment of CD4^+^ and CD8^+^ T lymphocytes, with enhanced pro-inflammatory responses participate in granuloma formation and parasite control. As a result, few parasites remain in the lesions and promote a delayed-type hypersensitivity (DTH) (**Figure 4**) (10, 93, 94). However, differences in intralesional immune profiles between the different CL forms are not well documented. Still, the infection outcome is clearly the consequence of a balance between pro and anti-inflammatory responses.

### In *L. major* infection

During *L. major* infection, promastigotes deposition in the skin promotes neutrophils and dendritic cells (DCs) recruitment during the earliest stage of infection (95). In this stage, Chaves et al. demonstrated the anti-inflammatory functions of dermal tissue-resident macrophages, which was supported by the reduced parasite burdens observed in mice (96). The clearance and control of *L. major* multiplication involved mainly cytotoxic CD8^+^ T cells and CD4^+^ helper T cells and the production of IFN-γ (90, 97); in fact, non-healing lesions in mice are associated with a small number of TCD4^+^ (98, 99). However, PWK mice strain develops prolonged but self-healing lesions, with an immune response characterized by a mixed Th1-plus-Th2 pattern acquiring resistance to a secondary challenge (100). Formaglio et al. recently showed the implication of the resident memory CD4^+^ T cells in delayed-hypersensitivity response, without the involvement of circulating T cells but with the recruitment of activated inflammatory monocytes. These monocytes produce reactive oxygen species (ROS) and nitric oxide (NO) that play a role in the inhibition of *L. major* development in the mice (101). These findings underscore the central role of skin-resident CD4^+^ T cells’ in enhancing the protective immune response against *L. major* (102). Independently of the T resident memory cells, the acute availability of circulating CD4^+^ T helper 1 effector cells (Th1EFF) at the time of secondary infection is critical for the Th1 immune response. Th1EFF cell-phagocyte interactions proved crucial in preventing the establishment of a permissive *L. major* niche in lesions (103). However, if NK cells may play several roles in developing an effective T cell response against *Leishmania*, their contributions to the response to CL in humans are less clear. For instance, it has been shown that NK-cell-derived IFN-γ, in mice, is essential for activating the dendritic cells that mediate the T-cell-dependent protection against *L. major* infection (104). Another mechanism associated with Human skin lesions protection is granzyme B−dependent CD8 T-cell cytotoxicity, as it participates in controlling parasite multiplication through a cytotoxic process (88, 105). On the other hand, in mice, a negative regulatory role for IL-4 was identified in limiting the recruitment of Th1 cells to *L. major* infected tissues, which alters the *Leishmania* clearance (89, 106). Therefore, the study of the impact of IL-4 on lesion evolution could provide hints for a therapeutic trial (107–109). In addition, the cytokine balance IL-4/IFN-γ has also been demonstrated to participate in the commitment of local immune response. Indeed, IL-4 secretion at the site of *L. major* infection rather than low IFN-γ production may play a role in the prolongation of disease during acute and chronic CL lesions. A moderate increase of CD4 Tregs would be observed in chronic lesions. However, few studies have suggested the role of Tregs during human CL; Belkaid et al. demonstrated that Tregs are rapidly accumulated at sites of *L. major* infection, favoring the early parasite expansion, contributing to the maintenance of immunological self-tolerance, and coinciding with the expression of effective immune responses (98, 110). In a group of *L.major* infected patients, Hoseini et al. observed a significantly higher expression of Foxp3 in chronic lesions compared to acute lesions; The moderate increase of T reg in chronic lesions and their function in persistent infection is still not apparent (111).

### In *L. infantum* infection

*L. infantum* is mostly responsible for visceral leishmaniasis (LV) but can also cause CL; little is known about the pathogenesis of this form. Recently, the level of INF-γ was evaluated for sporadic CL and zoonotic CL and was found to be significantly higher in *L. infantum* lesions than due to *L. major* (90). As mentioned above, for CL caused by *L. major*, CD8^+^ T cells are an essential part of the defense mechanisms against the parasite; however, for *L. infantum* infection, IFN-γ has a long-range effect inducing skin tissue destruction and keratinocyte apoptosis (10, 112, 113). In addition, it has been shown that increased IL4 production in skin lesions of *L. infantum-* infected dogs is associated with severe clinical signs and a high parasite burden (114). Although the role of IL4 was little described for *L. major* infection, no data was found for *L. infantum* human cutaneous Leishmaniasis. Furthermore, there is still much to be learned regarding the role of T reg cells on skin lesions in human CL caused by *L. infantum*; authors have suggested that T reg cells and the regulatory cytokines, especially TGF-*β*, play an essential role in the immunopathogenesis of non-typical ulcerated leishmaniasis (NUCL), modulating the cellular immune response in the skin, avoiding tissue damage, and leading to low parasitic persistence in the skin (115, 116). These factors could play a role in regulating the cellular immune response balance, resulting in the maintenance of a low tissue parasitism that avoids lesion growth.

### In *L. tropica* infection

Although little is known about the *in situ* immune status of *L. tropica*-infected patients, cases develop a chronic CL with a strong delayed-type hypersensitivity (117). It has been recently, shown that *L. tropica* has an enhanced capacity to reduce NO production by macrophages *in vitro*, which could help to understand why *L. tropica* infection could induce chronic lesions (118). In a study by Ajdary et al., evaluating T-cell responses to *Leishmania* antigen *in vitro*, Th2 cell response was dominant in active CL cases, and Th1 characterized the group of healed patients (119). The level of specific cytokine was evaluated *in vitro* in the PBMC but not in the skin, and high levels of IFN-γ, IL-5, and IL-13 in non-healing patients were observed, suggesting a mixed Th1/Th2 response with chronic lesions. In contrast, patients with acute lesions respond to infection *via* a Th1-type response (119). However, such a result must be confirmed locally in skin lesions or animal models. It has been discovered that in the early stages of *L. tropica* infection, the amount of IL-4 is upregulated and linked to a higher parasite burden. This may play a role in the development of CL by inhibiting the protective immune response. These findings indicate that the parasites may rapidly multiply at the beginning of the infection, leading to a Th2-type immune response (120). Information on regulatory immune responses is lacking for *L. tropica* CL. However, *L. tropica*-infected lesions from Indian patients, the analysis of localized immune response reveals the presence of Th17 and T reg cells (121). Because of the difficulties in establishing infection *in vivo*, published data using animal models for leishmaniasis caused by *L. tropica* is limited (122). Nevertheless, it was suggested that the presence of Tregs in infected tissues may be a possible host immune response or homeostatic mechanism to control *L. tropica* infection and to reduce the excessive inflammation supporting parasite survival through IL-10. Still, additional investigations are needed to clarify this response.

### In *L. braziliensis* infection

The severity of lesions that develop in patients infected by *L. braziliensis* is mainly associated with a highly inflammatory cutaneous environment. In fact, patients with *L. braziliensis* infection exhibit a strong T-helper 1 (Th1) immune response, which leads to excessive inflammation and tissue damage (123). High levels of both IFN-γ and TNF-α are observed in CL caused by this species. Still, while IFN-γ may have a protective function (124), there is strong evidence to support the role of TNF-α in the pathology of cutaneous and mucosal lesions (125–130). Thiago Cardoso and colleagues (2014) studied the protective and pathological functions of CD8^+^ T Cells in *L. braziliensis* infection. The frequency of CD8^+^ T cells expressing granzyme in the lesions of severe CL patients is more significant than that in patients during early stage of CL (131). However, cytotoxic CD8 T cells are harmful to both *L. braziliensis* and infected host cells because cytotoxicity is higher in mucocutaneous leishmaniasis than in the localized form (132, 133). Recently, it has been observed a high proportion of senescent T cells (Tsen) with high inflammatory profiles in *L. braziliensis* lesions with mucosal involvement (134, 135), which is linked to the severity and tissue damage (136). Therefore, *in vivo*, senescent CD8+ T cells appear to be the most important cell populations mediating skin pathology (137). Other cells, particularly Treg cells, accumulate in lesions caused by *L. braziliensis*, and *contribute* to the local control of effector T-cell functions (138). Campanelli et al. research suggests that Treg accumulated at the sites of *L. braziliensis infection* may contribute to the local control of effector T cell functions. Still, a direct correlation with the pathogenesis is yet to be detected (138).

### In *L. guyanensis* infection

**Figure 5:**
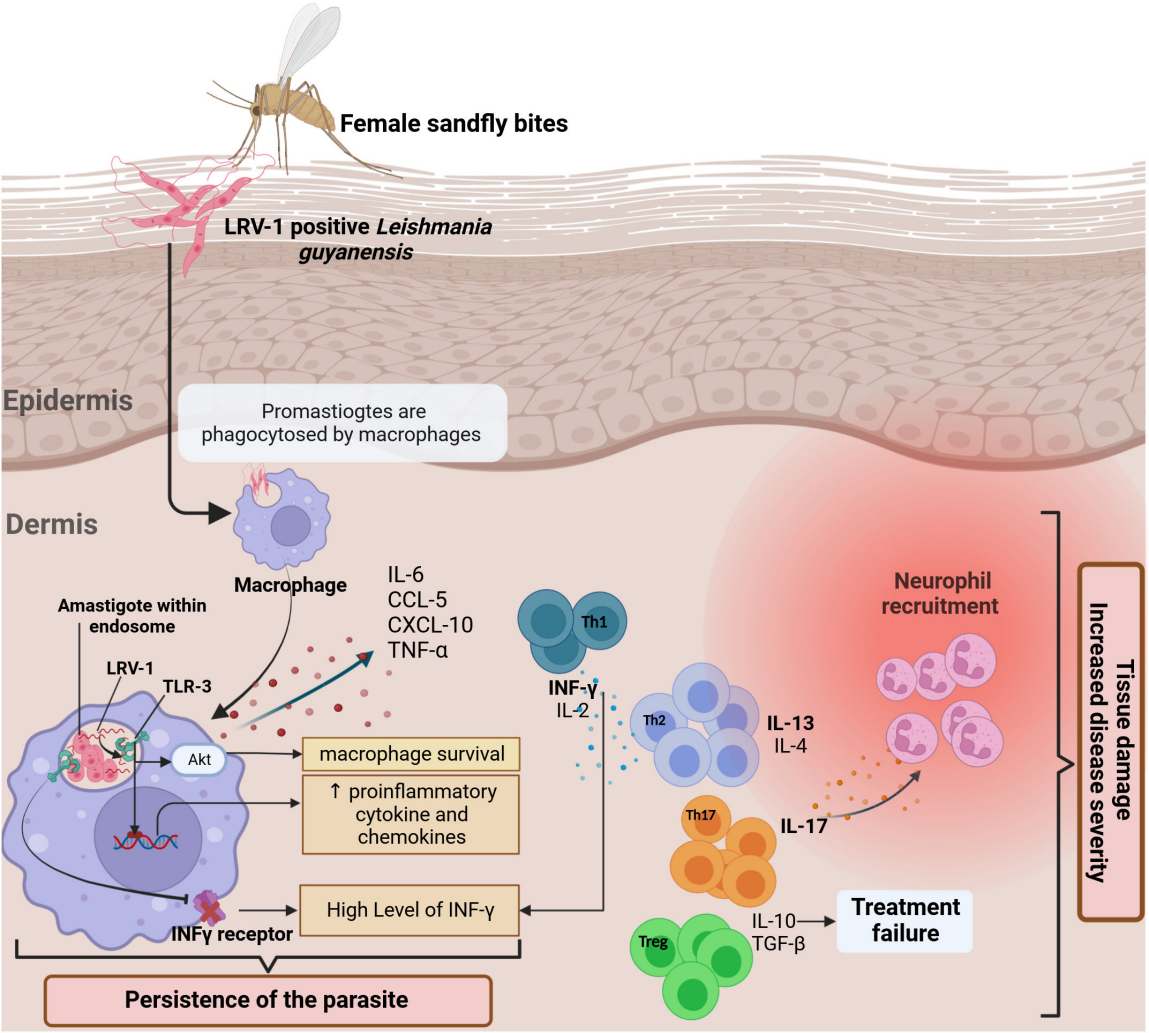
The *in situ* immune response against *L. guyanensis* contains a dsRNA virus called (LRV-1). The response to the virus is mediated by TLR 3 in the endosomal macrophage compartment, where the parasite lives and divides. A hypothesis is that after the infection, the viral RNA is released after parasite death and binds to TLR-3 (143), promoting pro-inflammatory cytokines and chemokines production such as IL-6, TNF-α, CXCL-10, and CCL-5 and controlling the severity of the disease (144). Generally, *L. guyanensis* infection induces a mixed Th1/Th2/Th17 immune response. Th-17 cells appear to predominate in lesions in the presence of *Leishmania* RNA with high production of IL17-A. TGF-β is essential for establishing infection and, together with IL-10, leads to therapeutic failures and increased disease severity. The stimulation of TLR3 results in the downregulating of IFN-γ receptor expression, reducing macrophage activation, which explains the high level of IFN-γ observed in lesions produced by Th-1. Indeed, *via* Akt (Protein kinase B) signaling, TLR3 activation by LRV1 promoted parasite persistence (145). Altogether, it enhances inflammation and thus exacerbates disease.

**Figure 6:**
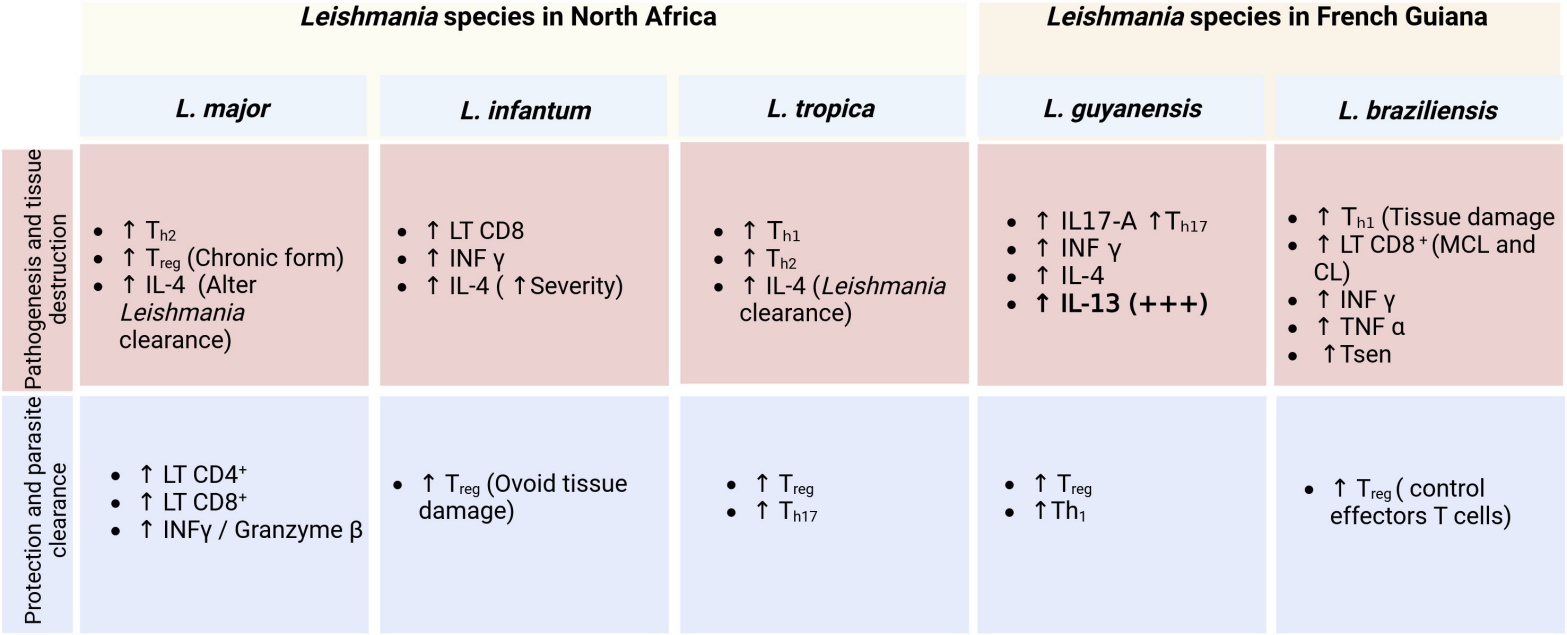
Comparison between the *in situ* immune profiles in lesions of patients infected with the five *Leishmania* species (*L. major, L. infantum, L. tropica, L. guyanensis, L. braziliensis*) and the implication for pathogenesis and the control of the diseases.

Little is known about the immune responses induced during human infection with *L. guyanensis*, which generally generates a mixed Th1/Th2/Th17 immune response. IL-13 is the main Th2 cytokine found in *L. guyanensis* LCL lesions, according to research by Bourreau and colleagues. As IL-13 has many similar effects with IL-4 (139), patients with *L. guyanensis* LCL are likely to have a Th2 response that includes the production of either IL-4 or IL-13. However, IL-13 plays a key role in maintaining the Th2 response in Human leishmaniasis by making certain cells resistant to IL-12 (140). In *L. guyanensis* infection, the levels of Foxp3 in the lesions were superior in chronic patients than in acute ones demonstrating the regulatory role of T reg. Furthermore, Tregs isolated from skin biopsy of *L. guyanensis* patients with acute CL had a suppressive effect on CD4+T effector (eff) cells. Also, Foxp3 expressions were higher in skin biopsies than in peripheral blood mononuclear cells (PBMC), confirming the recruitment of Tregs to the infection site (141, 142). *L. guyanensis* shows unique characteristics with a mixed immune response (**Figures 5**, **6**), which warrants further investigation to uncover the mechanisms that regulate immune responses and control inflammation caused by this parasite. Recently, a proposed relationship between *Leishmania* RNA Virus (LRV) and the severity of leishmaniasis has been suggested, as various factors are linked to the pathogenicity of the disease (146). Zabala-Peñafiel and colleagues demonstrated that *L. guyanensis* metastatic strains had a higher rate of LRV1 positivity than non-metastatic strains (147). During macrophage infection, it caused over-expression of pro-inflammatory mediators such as TNF-α and IL-6. Ginouvès and colleagues found that 74% of the *Leishmania* (*Viannia*) subgenus clinical isolates in French Guiana contain LRV1, with the majority being *L. braziliensis* and *L. guyanensis*. Patients infected with LRV1-positive *L. guyanensis* have a high ratio of IL-17A and IFNγ in their skin lesions (146) (**Figure 5**). This is accompanied by higher levels of IL-17A in blood cell cultures after stimulation with live parasites, compared to those infected with LRV1-free *L. guyanensis* (148). This demonstrates that virus infection can exacerbate atypical tegumentary leishmaniasis caused by *L. guyanensis*. These findings suggest that the presence of LRV1 in the parasites not only increases the risk of developing ML but also causes complications in CL. Contradictorily, in a study conducted by Ginouvès et al., no correlation was found between the presence of LRV1 or its genotypes in *L. guyanensis* parasites and treatment failure, either after the first or second course of treatment of pentamidine (149). Additional research is required to assess a possible link between LRV1-infected parasites and their clinical symptoms.

## Conclusion

The outcome of *Leishmania* infection relies on the intricate equilibrium of pro- and anti-inflammatory immune responses generated by the host. Both innate and adaptive immunity are inextricably linked to each other as the cytokines produced by cells of the innate system determine the outcome and magnitude of the adaptive immune response. Therefore, the immune responses need to be tightly regulated to avoid immune-mediated pathology to host tissue. Here, we have highlighted recent progress made in understanding the immune response to cutaneous leishmaniasis caused by *Leishmania major*, *L. tropica*, and *L. infantum* in North Africa on the one hand, and by *L. guyanensis* and *L. braziliensis in* French Guiana on the other hand. If cellular and cytokines are released in the lesion during OW-CL, much remains to be known in NW-CL. Indeed, this review could significantly contribute to understanding local immune parameters that control and protect the pathogenesis during *Leishmania* infection. However, a better knowledge of the local immune response in Human CL may help to identify new mechanisms and targets to develop new and more adapted treatments. New local therapies for cutaneous leishmaniasis in NA, French Guiana, and all endemic countries are urgently needed. Drug development is considered a priority, as most current treatments are based on expensive drugs or potential side effects. The cost of anti-Leishmanial drugs is a crucial issue in low-income countries. Besides, therapeutic failures remain frequent, and cases of unresolved CL are often reported, possibly because all current drugs only target the parasite, not the host responses. This review demonstrates how these responses can shape the clinical lesions and lead to different forms. Research efforts should focus on testing immunotherapies that could reduce the severity of pathology seen in some cases of cutaneous leishmaniasis, which could consecutively lessen the duration of antiparasitic courses and their toxicity. In the last decade, several drugs have been developed for other skin inflammatory diseases or as immunotherapy for skin cancers. Some of these drugs inhibit IL-4 or cytotoxicity and might be helpful in combination with existing anti-parasitic drugs. Anti-IL 4 drugs could be useful for all species of *Leishmania*, while specific inhibitors of IL-13 and IL-17 could be used to dampen tissue damage in *L. guyanensis* infections (**Figure 6**). Dupilumab is currently used in atopic dermatitis both for its IL-4 and IL-13 effects and could be tested in association with antiparasitic drugs. Other candidates could include anti-IL17 used for inflammatory skin diseases, such as secukinumab, brodalimumab or ixekizumab. Furthermore, skin dysbiosis has been shown to drive the inflammatory response in *L. major* infected germ-free mice model (150). These observations emphasize the need to explore mechanisms by which skin microbiota is involved in the physiopathology of CL. In our opinion, it is essential that these advances in the role of microbiota should be included in the research for the development of new drugs and vaccines against CL.

## Author contributions

SP designed, revised, and supervised the work. NS reviewed the literature, created the figures, and wrote the manuscript. RB, GP, KA, MD, PC, AB participated in the manuscript’s analysis, drafting, and revising. All authors revised and approved the manuscript’s final version.
